# Utilization of Platelet-Rich Plasma in Oral Surgery: A Systematic Review of the Literature

**DOI:** 10.3390/jcm14082844

**Published:** 2025-04-20

**Authors:** Andrea Giannelli, Marta Forte, Giuseppe D’Albis, Giulia Cianciotta, Luisa Limongelli, Laura Stef, Ramona Feier, Abdulrahman Omar Alrashadah, Massimo Corsalini, Saverio Capodiferro

**Affiliations:** 1 Private Practitioner, 73046 Matino, Italy; andreagiannel@gmail.com; 2 Department of Interdisciplinary Medicine, University of Bari “Aldo Moro”, Piazza Giulio Cesare, 11, 70124 Bari, Italy; cianciottagiulia20@gmail.com (G.C.); luisa.limongelli@uniba.it (L.L.); massimo.corsalini@uniba.it (M.C.); saverio.capodiferro@uniba.it (S.C.); 3 Faculty of Medicine, Lucian Blaga University Sibiu, 550169 Sibiu, Romania; laura.stef@ulbsibiu.ro; 4 Faculty of Medicine, “Dimitrie Cantemir” University, 700115 Iasi, Romania; dr.ramonafeier@yahoo.ro; 5 Department of Dentistry, King Faisal University, P.O. Box 380, Al Hofuf 31982, Saudi Arabia; dr.alrashadah@gmail.com

**Keywords:** PRP, growth factors, oral surgery, implantology, dentistry, oral health

## Abstract

**Introduction:** The physiological process of wound healing is a complex and dynamic series of events that aims to restore damaged tissues to their original structure and function. Platelet-rich plasma (PRP), an autologous blood-derived product, is characterized by a high concentration of platelets suspended in a small volume of plasma, along with a complete array of coagulation factors at physiological concentrations. Beyond platelets, PRP contains a significant quantity of bioactive growth factors, such as platelet-derived growth factor (PDGF), vascular endothelial growth factor (VEGF), and transforming growth factor-beta (TGF-β), all of which are crucial mediators of tissue repair and osteogenesis. Due to these properties, PRP has garnered considerable attention in oral surgery, where the efficient regeneration of both hard and soft tissues is critical for the optimal therapeutic outcomes. **Objectives:** This systematic review aimed to critically evaluate the efficacy of PRP in oral surgical procedures, with particular emphasis on its role in the regeneration of both soft and hard tissues, as well as its clinical outcomes. Furthermore, the review sought to identify the diverse surgical applications of PRP and assess the impact of its use in conjunction with grafting materials on regenerative outcomes. **Methods:** A comprehensive systematic review was conducted, analyzing articles published within the last decade regarding the application of PRP in oral surgery, specifically focusing on periodontal, regenerative, and implant-related procedures. Studies were selected based on rigorous inclusion criteria, assessing the utilization of PRP across different clinical settings. **Results:** Thirteen relevant studies were included, which were categorized as follows: three studies involving implant surgery, three studies focusing on third molar extractions, two studies on regenerative surgery, two studies addressing periodontal surgery, one study examining intrabony periodontal defects, and two studies on ridge augmentation procedures. The majority of studies reported modest improvements in clinical parameters such as periodontal probing depth and clinical attachment level (CAL). Furthermore, significant positive outcomes were observed in soft tissue healing, with notable enhancements in bone density. These results suggest that PRP may facilitate the healing process, particularly in soft tissues, while also promoting bone regeneration to a degree. **Conclusions:** The findings of this systematic review underscore the potential of PRP as a valuable adjunct in oral surgery, demonstrating significant benefits in the regeneration of soft tissues and, to a lesser extent, hard tissues. Notably, the standalone application of PRP did not yield substantial improvements in regenerative outcomes. However, when PRP was used in combination with grafting materials, more pronounced benefits were observed, indicating a synergistic effect that enhances both soft and hard tissue regeneration. These findings support the rationale for incorporating PRP into clinical practice, particularly in conjunction with grafting materials, to optimize patient outcomes in oral surgery. Further research, particularly involving larger sample sizes and long-term follow-ups, is necessary to fully elucidate the optimal clinical applications and mechanistic pathways of PRP in oral regenerative procedures.

## 1. Introduction

Platelet-rich plasma (PRP) has gained considerable attention in oral surgery due to its efficacy and ease of application in promoting, accelerating, and enhancing healing processes. Unlike other tissue grafts, which are often difficult to procure and may present immunological challenges or complications at the donor site, PRP offers a readily accessible and biocompatible alternative, facilitating tissue regeneration with minimal risk [1]. Derived from the patient’s own blood, PRP undergoes a centrifugation process to concentrate bioactive components. Owing to its autologous nature, it exhibits osteoconductive properties, facilitating cellular migration, proliferation, and matrix deposition while minimizing immunogenic risks [2,3]. The procedure entails a dual-step centrifugation process: an initial spin to fractionate blood components, followed by a secondary centrifugation to isolate and concentrate the PRP, ensuring optimal bioactive compound retention for therapeutic application [4].

Consequently, PRP eliminates the risks associated with cross-contamination, disease transmission, and immune reactions inherent to the use of allogeneic blood products [3]. Beyond its high platelet concentration, PRP also contains key inflammatory cells (e.g., monocytes) and an array of bioactive proteins, including platelet-derived growth factor (PDGF), transforming growth factor-β (TGF-β), vascular endothelial growth factor (VEGF), epidermal growth factor (EGF), insulin-like growth factor (IGF), and fibroblast growth factor (FGF). These molecular mediators play a pivotal role in hemostasis, graft stabilization, and neovascularization, thereby reducing postoperative bleeding and accelerating tissue integration [3,5]. Consequently, PRP eliminates the risks associated with cross-contamination, disease transmission, and immune reactions inherent to the use of allogeneic blood products. Beyond its high platelet concentration, PRP also contains key inflammatory cells (e.g., monocytes) and an array of bioactive proteins, including platelet-derived growth factor (PDGF), transforming growth factor-β (TGF-β), vascular endothelial growth factor (VEGF), epidermal growth factor (EGF), insulin-like growth factor (IGF), and fibroblast growth factor (FGF). These molecular mediators play a pivotal role in hemostasis, graft stabilization, and neovascularization, thereby reducing postoperative bleeding and accelerating tissue integration. Extensive research supports PRP’s potential in enhancing wound healing and minimizing postoperative complications, although some studies present conflicting findings regarding its overall efficacy [6]. PRP has been widely applied in implantology, periodontology, and oral surgery, where its use in periodontal regeneration and bone grafting has yielded promising outcomes in facilitating both soft tissue and bone healing [7].

PRP is defined as autologous plasma with a platelet concentration exceeding baseline level. The standard protocol involves collecting 10 mL of the patient’s blood via venipuncture, typically from the antecubital vein, immediately prior to the surgical procedure. The blood is then transferred into a tube containing an anticoagulant to prevent premature clotting. Anticoagulant agents such as sodium citrate, citrate dextrose-A (ACD-A), or citrate phosphate dextrose (CPD) chelate calcium ions, thereby inhibiting the coagulation cascade and enabling controlled centrifugation [3]. Various centrifugation protocols have been documented in the literature, broadly categorized into single-spin centrifugation, double-spin centrifugation, and selective blood filtration techniques, each yielding PRP formulations with distinct cellular compositions and biological properties [8]. The most widely utilized PRP preparation technique follows a two-step centrifugation process. The initial centrifugation, referred to as the “hard spin”, is performed at 2400 revolutions per minute (r.p.m.) for 10 min. This step stratifies the blood components within the collection tube, resulting in a distinct separation: red blood cells (RBCs) settle at the bottom, while the buffy coat—comprising white blood cells (WBCs) and platelets—accumulates above. The uppermost layer consists of platelet-poor plasma (PPP), a plasma fraction with a minimal platelet concentration. Following this phase, the yellow PPP layer is carefully aspirated using a 16-gauge catheter, leaving behind the RBC fraction. The sample then undergoes a second centrifugation step, termed the “soft spin”, at 3600 r.p.m. for 10 min. This final centrifugation refines the separation by concentrating platelets while further isolating them from white blood cells. The resulting PRP preparation yields a platelet concentration of approximately 1,000,000 platelets/µL within a 5 mL volume, significantly surpassing baseline platelet levels. To enhance its bioactivity, PRP is subsequently activated using 5 mL of 10% calcium chloride (CaCl) or thrombin, which triggers the degranulation of alpha granules and the subsequent release of essential growth factors and bioactive proteins, optimizing its regenerative potential [3,6,9].

The capacity of PRP to enhance tissue repair and amplify the innate regenerative potential of oral tissues has driven a surge in research efforts focused on evaluating its efficacy, safety, and clinical applications [10]. Beyond its well-documented role in modulating inflammation and accelerating tissue healing, PRP has also been associated with an intrinsic analgesic effect, contributing to a significant reduction in postoperative pain. This multifaceted therapeutic profile underscores its potential as a valuable adjunct in oral surgical interventions, enhancing both tissue regeneration and patient comfort [11,12].

### 1.1. Classification of Platelet Concentrates

Depending on the specific preparation protocols, variations in cellular composition, and structural organization, platelets can be processed through multiple methodologies, each yielding distinct biological and clinical properties: pure PRP (P-PRP), PRF, leukocyte-rich platelet-rich plasma (L-PRP), and plasma rich in growth factors (PRGF) (Table 1) [12,13,14,15,16,17,18].

### 1.2. Drug Interactions

To ensure a high-quality PRP preparation, strict criteria must be established. The donor patient must be in good health and should not be taking any analgesic medications, particularly nonsteroidal anti-inflammatory drugs (NSAIDs) or aspirin. These pharmacological agents inhibit cyclooxygenase (COX)-mediated oxygen consumption, thereby preventing proper platelet activation and aggregation [19]. Such drug-induced alterations can compromise PRP functionality, as NSAIDs interfere with the physiological release of growth factors essential for tissue regeneration. Therefore, it is strongly recommended to collect the blood sample before any NSAID or aspirin intake to prevent platelet dysfunction and preserve the full therapeutic potential of PRP [20].

This systematic review aims to critically assess the efficacy of PRP in oral surgical procedures, with a focus on its regenerative potential in both soft and hard tissues, as well as its clinical outcomes. Furthermore, the authors comprehensively analyze the various surgical applications of PRP and explore its effects when used in conjunction with grafting materials, providing insights into its potential to enhance tissue healing and regeneration.

## 2. Materials and Methods

To perform this study, first research on MEDLINE (PubMed) and Cochrane library was performed. Also, authors used a web-tool, RAYYAN, to include or exclude studies. A detailed search strategy was crafted based on the PICO criteria, and the PICO question to respond was “Has PRP had positive outcomes in oral surgery?”; this current search utilized a combination of keywords and Medical Subject Headings (MeSH) terms, including: (((prp) OR (“platelet rich plasma”)) AND (oral surgery) AND (dentistry)) Filters: Full text, English, from 2013 to 2023. Also, a manual investigation was made through the article’s bibliography; books and other research were conducted by internal sources that authorized this investigation. Table 2 summarizes the workflow based on the PICO criteria.

### 2.1. Study Selection

Studies considered eligible for this review were selected from the last 10 years, in the field of implantology, periodontology and oral surgery, only written in the English language. To determine whether the articles found were able to be assessed in the study, it was given as a guideline that the word ‘PRP’ should have been written at least in the title and in the abstract. Also, clinical cases in which less than 3 patients were evaluated were excluded.

#### 2.1.1. Inclusion Criteria

1. Articles from the last 10 years
2. Main topic “oral surgery”
3. Presence of healthy or non-healthy periodontium
4. Implant surgery
5. Periodontal surgery
6. Articles written in English

#### 2.1.2. Exclusion Criteria

1. Systematic reviews
2. Reviews on animals
3. Case control
4. Meta-analyses
5. Medical fields not associated with oral surgery
6. PRP used in temporo-mandibular joint disorders
7. PRP used in cleft palate surgeries
8. Patient treated with sinus lifting [6,9,21,22,23,24,25,26,27,28,29,30,31]

### 2.2. Data Extraction

Data extracted to perform this review were as follows: author, name of the journal, gender, age, study design, prospective/retrospective study, number of males and females, treatment modalities, follow-up time, type of grafts, type of anticoagulant, anesthetic techniques, incisional approach, and centrifugation parameters (RPM settings).

### 2.3. Protocol and Registration

The protocol for the current study was registered with the International Prospective Register of Systematic Review (PROSPERO)—Registration number: 645254.

## 3. Results

### 3.1. Number of Studies

Comprehensive research identified 324 articles, although 103 articles were immediately excluded. A total of 212 studies were screened, and 135 were excluded after reading the abstract. From 212 studies, only 32 were assessed as eligible, and 18 articles were excluded because of the exclusion criteria; consequently, the final number of elected studies for this systematic review was 13.

**Figure 1:**
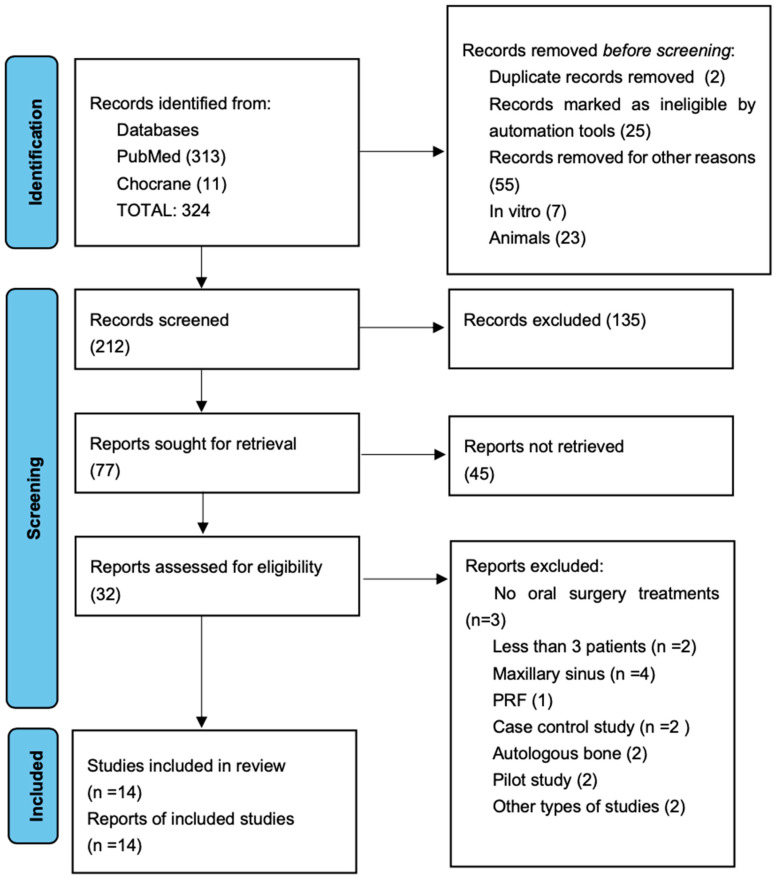
Flow chart of selected studies [32].

Figure 1 summarizes the flow chart. The main characteristics of the included articles are presented in Table 3. To perform this systematic review, 13 studied from the last 10 years were analyzed: in 3 studies, PRP was used in implant surgery [21,22,23], 3 in third molar extraction surgeries [6,9,24], 2 in regenerative surgery [25,26], 2 in periodontal surgery [27,28], 1 in intrabony periodontal defects [29], and 2 in ridge augmentation [30,31,32]. The assessed studies were prevalently randomized clinical trial and with a follow up of medium–long range from 16 weeks up to 5 years. In all the studies, the number of examined patients was always more than 5, with a total of 468 patients and overall average of 34, 28 patients per each study, with a prevalence of the female gender. Among the studies, only 2 of them reported the utilization of citrate phosphate dextrose adenine as the anticoagulant used [6,9]; the rest of the studies used sodium citrate. In all the test groups, PRP was used alone or combined with bone grafts like Bio-Oss or demineralized freeze-dried bone allograft (DFDBA). Also, in all surgical procedures, a full-thickness mucoperiosteal flap was executed.

**Figure 2:**
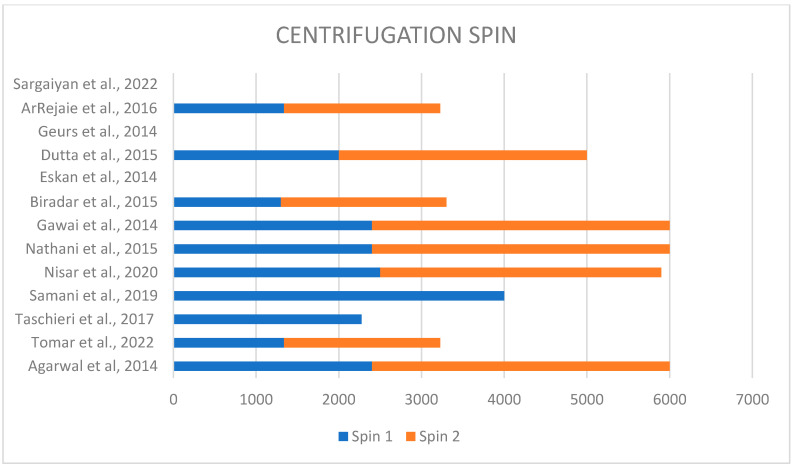
Different centrifugation speeds used in each study [6,9,21,22,23,24,25,26,27,28,29,30,31].

**Figure 3:**
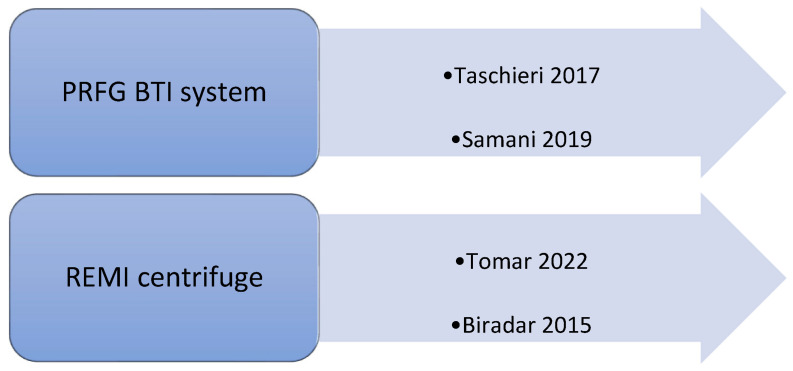
Types of centrifuges used in the selected studies [21,22,27,28].

Each study performed a different technique to obtain plasma rich in proteins. In fact, different techniques used different speeds of centrifugation, varying from 1300 rpm to 4000 rpm for the first spin and from 1890 rpm to 3600 rpm for the second spin (Figure 2). Only four studies discussed the type and brand of used centrifuge; in two of them, the PRFG BTI system was used [22,27]; in two cases, the REMI centrifuge was used [21,28] (Figure 3).

### 3.2. Outcomes

The investigated studies resulted in 57.14% of the procedure with positive outcomes in both soft and hard tissues. A total of 42.86% demonstrated an improvement in soft tissue healing and regeneration. Also, 42.86% showed an improvement in bone regeneration and bone density. It was assessed that when PRP was added to another graft material, the outcomes had better results compared to other graft materials used alone.

Only two articles had negative outcomes, since no influential differences were reported in the statistical analysis [21], and in one of them, PRP was compared to two other graft materials, synthetic allograft (PerioGlass) and bioresorbable xenograft (Bio-Oss), showing no advantages over these [21].

Overall, the negative outcomes were only observed in implant and periodontal surgeries; other studies had positive outcomes, in particular regarding soft tissue healing. Comparing the two groups, test and control, the results were considered significant when *p*-value < 0.05.

#### 3.2.1. Probing Depths

Three studies did not demonstrate any significant statistical changes in probing depth between test and control group after one year from the surgical procedure (*p* > 0.05) [21]. The other two studies regarding implant placement and regeneration of the periodontal defect after a third molar surgery were statistically significant (*p* < 0.05) [28,29] (Table 4).

#### 3.2.2. Clinical Attachment Level

Pooled data from two different studies showed statistically significant results in clinical attachment loss (CAL) measurement [28,29] (Table 5).

#### 3.2.3. Bone Density

Collected data in four studies showed that bone density reported after a follow-up period of 1 month, 4 months and 12 months, according to the studies, had significant results in three studies [9,23,24] (*p* < 0.05). (Table 6).

#### 3.2.4. Soft Tissue Healing

Three out of four articles showed statistically significant improvements in soft tissue healing (*p* < 0.05) [9,22,24,27], once their follow-up period was completed. The wound was examined after 7 or 14 days depending on the technique performed (Table 7).

#### 3.2.5. Bone Loss

Bone loss was also assessed in two different studies that analyzed the mesial (Table 8) and distal (Table 9) portion after a follow up lasting from 3 to 12 months, showing non-statistically difference in one study (*p* > 0.05) [21]. The study in which PRP was combined with xenograft showed statistically significant results (*p* < 0.05) [23].

## 4. Discussion

### 4.1. Results Analysis

Over the past two decades, platelet-rich plasma (PRP) has emerged as a versatile biomaterial widely employed across various medical disciplines, including dentistry. Its therapeutic potential is attributed to the high concentration of bioactive growth factors it contains, such as platelet-derived growth factor (PDGF), epidermal growth factor (EGF), vascular endothelial growth factor (VEGF), and transforming growth factor-beta (TGF-β). These factors play critical roles in modulating cellular proliferation, tissue regeneration, and angiogenesis, making PRP a valuable adjunct in promoting healing and enhancing clinical outcomes [3].

This systematic review examined the efficacy of PRP across various surgical procedures, including periodontics, implantology, third molar extractions, and ridge augmentation, focusing on its regenerative potential. Fourteen studies comparing PRP-treated groups to controls (either without PRP or using alternative grafts) were analyzed. However, heterogeneity in methodologies and reported data limited comparability. Outcomes assessed—such as probing depth, clinical attachment levels (CALs), bone density, and soft tissue healing—varied across studies, and not all parameters were consistently measured. Despite these limitations, PRP showed promising effects on both soft and hard tissue regeneration, with significant improvements in probing depth and CALs, supporting its potential role in periodontal healing. However, further investigation is needed to fully delineate its efficacy in these specific clinical parameters [21,28,29]. These data yielded positive outcomes following long-term follow-up, which facilitated a more comprehensive analysis and highlighted sustained improvements. However, other studies failed to demonstrate significant enhancements with PRP application across various treatments, resulting in negligible differences between test and control groups. This variability underscores the need for further investigation to clarify the conditions under which PRP may exert its therapeutic benefits [21,24]. However, some discrepancies were noted, as Biradar et al. suggested that there were insignificant differences after a 14-day follow up [28]. Moreover, the use of PRP alone was compared with others graft materials, revealing minimal differences in outcomes [21].

Overall, the use of PRP yielded promising results in terms of bone density, with significant statistical improvements observed. Additionally, when outcomes were evaluated after extended periods, allowing adequate time for bone regeneration, the evidence indicated a notably accelerated regenerative process. These findings suggest that PRP may enhance both the speed and quality of bone healing, particularly when given sufficient time for the tissue to mature and remodel [9,23,24].

Periodontal disease involves two types of tissues: soft tissues and hard tissues. While PRP does not appear to directly influence bone regeneration, some evidence suggests that it can enhance the healing of intrabony periodontal defects. PRP may promote the regeneration of soft tissue structures and contribute to the stabilization and restoration of the periodontal attachment, thereby improving clinical outcomes in cases of periodontal disease [29]. As a result, PRP demonstrates a distinctly beneficial impact on soft tissues across various types of surgical procedures. Its bioactive components promote enhanced healing, reduce inflammation, and accelerate tissue regeneration, making it a valuable adjunct in soft tissue repair, regardless of the surgical context [9,22,27]. Gawai et al. noted significant results in tissue healing compared to control groups [6]. In contrast, Sargaiyan et al. reported that PRP did not yield favorable results in soft tissue healing, typically assessed after 7 or 14 days. This discrepancy is likely attributable to the small sample size of patients included in the study, which may have limited the statistical power to detect meaningful differences in healing outcomes [24].

Other outcomes investigated, such as bone loss at the mesial and distal sites, did not reveal significant differences. Tomar et al. found no notable differences when PRP was compared with other grafting materials [21], while other studies reported improvements when PRP was combined with additional grafting materials [23]. Specifically, Tomar et al. demonstrated that PRP did not reduce bone loss compared to other grafting materials, suggesting that the efficacy of PRP in addressing bone resorption may be limited in certain contexts [21,22,23].

Growth factors included in PRP, such as PDGF and TGF, induce bone healing. Gawai et al., who used this material after a third molar extraction, demonstrated that it has positive results, although only positive in the first month, as the results were insignificant after four months [6].

In regenerative surgery, improvements in bone width are often overlooked, yet positive results have been observed in some studies [30]. Conversely, Nisar et al. reported positive outcomes in the height of crestal bone but found no significant changes in bone width. The lack of significant findings in bone width could likely be attributed to the suturing technique employed in their study, which may have influenced the overall regenerative process [25].

Additionally, when implants are placed, factors such as bone density and marginal bone loss should be thoroughly evaluated to achieve optimal osseointegration. The use of PRP has demonstrated positive results concerning these factors [9,22,23,27]. In clinical tests involving post-extraction implants, Taschieri et al. observed that PRP, when used in conjunction with implants placed in fresh extraction sockets, positively impacted soft tissue recovery [22]. This improvement in soft tissue healing is attributed to PRP’s stimulation of angiogenesis and collagen synthesis. Samani et al. [27] conducted a study comparing the healing process of the donor site following a free gingival graft (FGG), dividing subjects into two groups: one receiving PRP and the other not. Their findings revealed accelerated healing in the PRP-treated group, with wound closure occurring after one week compared to two weeks in the control group. Moreover, in implant surgeries, PRP demonstrated superior soft tissue outcomes compared to hard tissue outcomes, as observed when compared to control groups [22]. While various centrifugation methods can influence outcomes, particularly through differences in revolutions per minute, not all studies support this view. Most studies reported positive outcomes with minimal variation across different centrifugation settings. In one study, ArRajaie et al. employed cone–beam tomography to assess bone density and other potential improvements, an approach not utilized in other studies, providing a promising alternative for evaluating results [23]. All studies examining the combined use of PRP and grafting materials reported highly favorable outcomes compared to when these materials were used independently [25,26,29]. Geurs et al. demonstrated that PRP accelerated bone graft turnover [31], although other studies did not observe superior results with PRP in combination with grafting materials. Nevertheless, when PRP is paired with grafting materials, both regeneration rates and clinical outcomes are enhanced [33,34,35].

### 4.2. Limitations

The primary limitation of this study lies in the lack of consensus within the existing literature. Although the procedure was consistently performed using the same protocol, only a limited number of studies addressed specific characteristics that were not consistently reported across all papers. While certain studies included a sufficiently large sample size, it is recommended that future studies include no fewer than 20 patients. Given the limitations highlighted in this review, further investigation is needed to better understand the long-term effects of PRP in clinical practice. Additionally, not all studies provided detailed information regarding blood characteristics or whether patients had used anti-inflammatory medications, which may influence outcomes. Notably, when PRP was combined with grafting materials, the results were notably positive. As such, it is imperative to conduct further studies that compare cases involving the combined use of PRP and grafting materials over an extended follow-up period to better elucidate the full therapeutic potential of these interventions.

## 5. Conclusions

Whether employed as autologous biomaterial, platelets represent a biologically active reservoir of growth factors, owing to their endogenous origin and regenerative potential. Their clinical utility, particularly in oral surgery, has been well-described, PRP currently serving as a principal adjunct to facilitate and expedite wound healing. Furthermore, the adjunctive use of PRP in combination with grafting materials has demonstrated a capacity to enhance tissue regeneration and improve overall clinical outcomes. Evidence from the present study suggests that the local application of PRP during implant surgery may significantly accelerate the healing of both hard and soft peri-implant tissues. Nevertheless, further comparative investigations are required to delineate the differential outcomes of PRP used in isolation versus its combined application with other grafting substrates, thereby refining its optimal clinical indications.

## Figures and Tables

**Table 1:** Different types of platelets concentrate, according to their preparations [12,13,14,15,16,17,18].

Classification of Platelet Preparation		Description
**P-PRP**	Pure PRP	Prepared without leukocytes
**L-PRP**	Leukocyte-rich platelet-rich plasma	High density of platelets in plasma
**P-PRF**	Pure platelet-rich fibrin	High-density fibrin network
**L-PRF**	Leukocytes and PRF	Leukocytes and high-density fibrin network
**PRGF**	Plasma rich in growth factors	Red blood cells and white blood cells are eliminated

**Table 2:** Research question and PICO workflow summary.

Focused Question	Has PRP Had Positive Outcomes in Oral Surgery?
**PICO criteria**	
**Population**	Patients treated with PRP for implantology, periodontology or oral surgery
**Intervention or Exposure**	Electronic research searches: (((prp) OR (“platelet rich plasma”)) AND (oral surgery) AND (dentistry))
**Comparison**	Surgical techniques with no utilization of PRP
**Outcome**	Effectiveness of the intervention (complete healing), healing times, recurrence rates, complications (bleeding, infection, tissue damage), post-operative pain, patient satisfaction.

**Table 3:** Data extraction table.

Author (Year)	Study Design	Patients (M/F)	Treatment	Test Group	Controlled Group	Follow Up	Results
Biradar et al. [28] (2015)	prospective	30	Periodontal surgery	15 (PRP)	15 (CAF)	16 weeks	No significant difference between PRP+CAF outcome and only CAF outcome, but earlier healing when PRP was used
Samani et al. [27] (2019)	prospective	10	Periodontal surgery	10 (PRP)	10 (FGG)	2 months	PRP accelerated the healing process of gingival tissue wounds
ArRejaie et al. [23] (2016)	prospective	16	Implant surgery	16 (PRP + xenograft)	16 (xenograft)	12 months	PRP in conjunction with xenograft had better outcomes than xenograft alone, when used for treatment of dehiscence defects around an immediate dental implant
Taschieri et al. [22] (2017)	retrospective	109 (49/54)	Implant surgery	71 (PRP)	38 (NO PRP)	5 years	Significant soft tissue healing after 3 and 7 days after surgery of P-PRP test group
Tomar et al. [21] (2022)	prospective	90	Implant surgery	30 (PRP)	30 (xenograft) 30 (allograft)	12 months	No significant difference in grafting materials that were used in immediate implant procedures
Nathani et al. [26] (2015)	prospective	10 (9/1)	Regenerative surgery	N/A	N/A	4 months	Less pain, better soft tissue healing and better bone healing
Nisar et al. [31] (2020)	prospective	30	Regenerative surgery	N/A	N/A	6 months	PRP added to collagen plug provided a good socket preservation, good for the height, but no significant results for the width
Dutta et al. [9] (2015)	prospective	60 (29/31)	3rd molar surgery	30 (PRP)	30 (NO PRP)	4 months	Good soft and hard tissue regeneration
Gawai et al. [6] (2014)	prospective	5 (2/3)	3rd molar surgery	5 (PRP)	5 (NO PRP)	12 months	PRP enhanced bone healing and formation the 1 month. After 4 months there’s no added benefit, it improves the soft tissue healing
Sargaiyan et al. [24] (2022)	prospective	15 (7/8)	3rd molar surgery	15 (PRP)	15 (NO PRP)	2 months	PRP aided the bone and soft tissue healing
Eskan et al. [30] (2014)	prospective	28 (14/14)	Ridge augmentation	14 (PRP)	14 (CAN)	4 months	PRP enhanced bone regeneration and increased horizontal bone
Geurs et al. [31] (2014)	prospective	41 (12/29)	Ridge augmentation	12 (PRP, FDBA, TCP, collagen plug)	9 (Collagen plug)	2 months	PRP inclusion sped up the turnover of bone grafts.
Agarwal et al. [29] (2014)	prospective	24 (10/14)	Intrabony periodontal defect	24 (PRP/DFDBA)	24 (DFDBA/saline)	12 months	Statistically significant changes in bone density when PRP and DFDBA was used

PRP: platelet-rich plasma; CAN: cancellous allograft; FDBA: freeze dried bone allograft; TCP: tricalcium phosphate; FGG: free gingival graft; CAF: coronally advance flap; N/A: not available.

**Table 4:** Probing depth (in mm; mean ± Sd).

	Test				Control				Follow Up	*p*-Value
	Baseline				Baseline					
Study	Mean	Sd	Mean	Sd	Mean	Sd	Mean	Sd		
Biradar 2015 [28]	2.27	0.62	1.37	1.9	2.27	0.37	1.4	0.21	16 weeks	<0.05
Agarwal 2014 [29]	7.23	0.79	3.58	0.41	7.25	0.77	3.60	0.53	12 months	<0.001
Tomar 2022 [21]	-	-	2.16	0.24	-	-	2.14	0.24	12 months	>0.05

**Table 5:** Clinical attachment level (in mm: mean ± Sd).

			Test				Control		Follow Up	*p*-Value
	Baseline				Baseline					
Study	Mean	Sd	Mean	Sd	Mean	Sd	Mean	Sd		
Biradar 2015 [28]	4.99	0.78	1.81	0.78	5.08	0.90	1.91	0.89	16 weeks	<0.05
Agarwal 2014 [29]	8.42	0.73	5.27	0.57	8.44	0.76	6.04	0.57	12 months	<0.001

**Table 6:** Bone density.

	Test				Control				Follow Up	*p*-Value
	Baseline				Baseline					
Study	Mean	Sd	Mean	Sd	Mean	Sd	Mean	Sd		
Sargaiyan 2022 [24]	128.63	0.65	133.58	0.81	140.91	1.23	145.38	0.83	1 month	<0.05
Dutta 2015 [9]	−0.10	0.305	1.90	0.305	−1.60	0.770	0.27	0.640	3rd week–4 months	0.000
ArRejaie 2016 [23]	130.39	3.28	129.34	3.29	123.89	4.32	106.46	3.13	3 months–12 months	<0.05

**Table 7:** Soft tissue healing.

	Test				Control				Follow Up	*p*-Value
	Baseline				Baseline					
Study	Mean	Sd	Mean	Sd	Mean	Sd	Mean	Sd		
Taschieri 2017 [22]	4.7	0.49	4.85	0.36	4.85	0.36	3.75	0.44	3–7 days	<0.001
Sargaiyan 2022 [24]	3.08	0.77	4.75	0.34	3.0	0.73	4.6	0.54	1–7 days	>0.05
Dutta 2015 [9]	−0.13	0.346	4.53	0.571	−1.73	0.450	3.90	0.845	3–14 days	<0.05
Samani 2019 [27]	2.20	0.42	5.00	0.00	1.00	0.00	4.00	0.00	2–14 days	<0.001

**Table 8:** Bone loss mesial site (in mm).

	Test				Control				Follow Up	*p*-Value
	Baseline				Baseline					
Study	Mean	Sd	Mean	Sd	Mean	Sd	Mean	Sd		
Tomar 2022 [21]	5.01	0.32	3.09	0.32	4.88	0.345	3.14	0.36	3–12 months	>0.05
ArRejaie 2016 [23]	1.66	0.24	0.80	0.24	2.27	0.45	1.60	0.26	3 months–12 months	<0.05

**Table 9:** Bone loss distal site (in mm).

	Test				Control				Follow Up	*p*-Value
	Baseline				Baseline					
Study	Mean	Sd	Mean	Sd	Mean	Sd	Mean	Sd		
Tomar 2022 [21]	4.87	0.34	3.25	0.36	4.15	0.345	3.175	0.355	3–12 months	>0.05
ArRejaie 2016 [23]	1.76	0.24	0.82	0.71	2.17	0.41	1.50	1.06	3 months–12 months	<0.05

## Data Availability

The data collected in the current study were downloaded from the following databases: PubMed (https://pubmed.ncbi.nlm.nih.gov; URL accessed on 31 December 2023), Scopus (https://www.scopus.com; URL accessed on 2 January 2024), and Web of Science (https://clarivate.com/academia-government/scientific-and-academic-research/research-discovery-and-referencing/web-of-science/; URL accessed on 3 January 2024).

## References

[1] Mihaylova Z., Mitev V., Stanimirov P., Isaeva A., Gateva N., Ishkitiev N. (2017). Use of platelet concentrates in oral and maxillofacial surgery: An overview. Acta Odontol. Scand..

[2] Rughetti A., Giusti I., D’Ascenzo S., Leocata P., Carta G., Pavan A., Dell’Orso L., Dolo V. (2008). Platelet gel-released supernatant modulates the angiogenic capability of human endothelial cells. Blood Transfus..

[3] Marx R.E. (2001). Platelet-Rich Plasma (PRP): What Is PRP and What Is Not PRP?. Implant. Dent..

[4] Saqlain N., Mazher N., Fateen T., Siddique A. (2023). Comparison of Single and Double Centrifugation Methods for Preparation of Platelet-Rich Plasma (PRP). Pak. J. Med. Sci..

[5] Schliephake H. (2002). Bone growth factors in maxillofacial skeletal reconstruction. Int. J. Oral Maxillofac. Surg..

[6] Gawai K.T., Sobhana C.R. (2015). Clinical Evaluation of Use of Platelet Rich Plasma in Bone Healing. J. Maxillofac. Oral Surg..

[7] Feigin K., Shope B. (2019). Use of Platelet-Rich Plasma and Platelet-Rich Fibrin in Dentistry and Oral Surgery: Introduction and Review of the Literature. J. Vet. Dent..

[8] Muthuprabakaran K., Pai V.V., Ahmad S., Shukla P. (2021). A cross-sectional analysis of the effects of various centrifugation speeds and inclusion of the buffy coat in platelet-rich plasma preparation. Indian. J. Dermatol. Venereol. Leprol..

[9] Dutta S.R., Singh P., Passi D., Patter P. (2015). Mandibular Third Molar Extraction Wound Healing With and Without Platelet Rich Plasma: A Comparative Prospective Study. J. Maxillofac. Oral. Surg..

[10] Aghaloo T.L., Moy P.K., Freymiller E.G. (2002). Investigation of platelet-rich plasma in rabbit cranial defects: A pilot study. J. Oral Maxillofac. Surg..

[11] Del Fabbro M., Corbella S., Ceresoli V., Ceci C., Taschieri S. (2015). Plasma Rich in Growth Factors Improves Patients’ Postoperative Quality of Life in Maxillary Sinus Floor Augmentation: Preliminary Results of a Randomized Clinical Study. Clin. Implant. Dent. Relat. Res..

[12] Del Fabbro M., Ceresoli V., Lolato A., Taschieri S. (2012). Effect of Platelet Concentrate on Quality of Life after Periradicular Surgery: A Randomized Clinical Study. J. Endod..

[13] Ehrenfest D.M.D., Andia I., Zumstein M.A., Zhang C.Q., Pinto N.R., Bielecki T. (2014). Classification of platelet concentrates (Platelet-Rich Plasma-PRP, Platelet-Rich Fibrin-PRF) for topical and infiltrative use in orthopedic and sports medicine: Current consensus, clinical implications and perspectives. Muscles Ligaments Tendons J..

[14] Dohan Ehrenfest D.M., Rasmusson L., Albrektsson T. (2009). Classification of platelet concentrates: From pure platelet-rich plasma (P-PRP) to leucocyte- and platelet-rich fibrin (L-PRF). Trends Biotechnol..

[15] Pavlovic V., Ciric M., Jovanovic V., Stojanovic P. (2016). Platelet Rich Plasma: A short overview of certain bioactive components. Open Med..

[16] Nishiyama K., Okudera T., Watanabe T., Isobe K., Suzuki M., Masuki H., Okudera H., Uematsu K., Nakata K., Kawase T. (2016). Basic characteristics of plasma rich in growth factors (PRGF): Blood cell components and biological effects. Clin. Exp. Dent. Res..

[17] Anitua E., Sánchez M., Orive G., Andía I. (2007). The potential impact of the preparation rich in growth factors (PRGF) in different medical fields. Biomaterials.

[18] Sun J., Hu Y., Fu Y., Zou D., Lu J., Lyu C. (2022). Emerging roles of platelet concentrates and platelet-derived extracellular vesicles in regenerative periodontology and implant dentistry. APL Bioeng..

[19] Moncada S.V.J. (1979). Arachidonic acid metabolites and the interactions between platelets and blood-vessel walls. N. Engl. J. Med..

[20] Schippinger G., Prüller F., Divjak M., Mahla E., Fankhauser F., Rackemann S., Raggam R.B. (2015). Autologous Platelet-Rich Plasma Preparations: Influence of Nonsteroidal Anti-inflammatory Drugs on Platelet Function. Orthop. J. Sports Med..

[21] Tomar N., Dahiya S., Sharma P.K., Parihar A.S., Mandal A., Sahoo A.R., Manas A., Parihar A.A.S., Anuradha B. (2022). A Comparative Clinical Evaluation of Effectiveness of Platelet-Rich Plasma, Synthetic Allograft, and Bioresorbable Xenograft During Immediate Implant Placement. Cureus.

[22] Taschieri S., Lolato A., Ofer M., Testori T., Francetti L., Del Fabbro M. (2017). Immediate post-extraction implants with or without pure platelet-rich plasma: A 5-year follow-up study. Oral Maxillofac. Surg..

[23] ArRejaie A., Al-Harbi F., Alagl A., Hassan K. (2016). Platelet-Rich Plasma Gel Combined with Bovine-Derived Xenograft for the Treatment of Dehiscence Around Immediately Placed Conventionally Loaded Dental Implants in Humans: Cone Beam Computed Tomography and Three-Dimensional Image Evaluation. Int. J. Oral Maxillofac. Implant..

[24] Sargaiyan V., Manas A., Hemanth Kumar H., Saravanan M., Ghiaz K., Deepalakshmi S. (2022). The role of prp in third molar extraction wounds: A clinical study. J. Pharm. Bioallied Sci..

[25] Nisar N., Nilesh K., Parkar M.I., Punde P. (2020). Extraction socket preservation using a collagen plug combined withplatelet-rich plasma (PRP): A comparative clinico-radiographic study. J. Dent. Res. Dent. Clin. Dent. Prospect..

[26] Nathani D., Sequeira J., Rao B.S. (2015). Comparison of platelet rich plasma and synthetic graft material for bone regeneration after third molar extraction. Ann. Maxillofac. Surg..

[27] Samani M.K., Saberi B.V., Ali Tabatabaei S.M., Moghadam M.G. (2017). The clinical evaluation of platelet-rich plasma on free gingival graft’s donor site wound healing. Eur. J. Dent..

[28] Biradar S., Satyanarayan A., Kulkarni A., Patti B., Mysore S., Patil A. (2015). Clinical evaluation of the effect of platelet rich plasma on the coronally advanced flap root coverage procedure. Dent. Res. J..

[29] Agarwal A., Gupta N.D. (2014). Platelet-rich plasma combined with decalcified freeze-dried bone allograft for the treatment of noncontained human intrabony periodontal defects: A randomized controlled split-mouth study. Int. J. Periodontics Restor. Dent..

[30] Eskan M.A., Qreenwell H., Hill M., Morton D., Vidal R., Shumway B. (2014). Platelet-Rich Plasma-Assisted Guided Bone Regeneration for Ridge Augmentation: A Randomized, Controlled Clinical Trial. J. Periodontol..

[31] Geurs N., Ntounis A., Vassilopoulos P., Van Der Velden U., Loos B.G., Reddy M. (2014). Using Growth Factors in Human Extraction Sockets: A Histologic and Histomorphometric Evaluation of Short-Term Healing. Int. J. Oral Maxillofac. Implant..

[32] Page M.J., McKenzie J.E., Bossuyt P.M., Boutron I., Hoffmann T.C., Mulrow C.D., Shamseer L., Tetzlaff J.M., Akl E.A., Brennan S.E. (2021). The PRISMA 2020 statement: An updated guideline for reporting systematic reviews. Bmj.

[33] Bezerra B.T., Pinho J.N.A., Figueiredo F.E.D., Brandão J.R.M.C.B., Ayres L.C.G., da Silva L.C.F. (2019). Autogenous Bone Graft Versus Bovine Bone Graft in Association with Platelet-Rich Plasma for the Reconstruction of Alveolar Clefts: A Pilot Study. Cleft Palate Craniofac. J..

[34] Marx R.E., Carlson E.R., Eichstaedt R.M., Schimmele S.R., Strauss J.E., Georgeff K.R. (1998). Platelet-rich plasma: Growth factor enhancement for bone grafts. Oral Surg. Oral Med. Oral Pathol..

[35] United Nations Department of Economic and Social Affairs (2023). The Sustainable Development Goals Report 2023: Special Edition [Internet]. United Nations. (The Sustainable Development Goals Report). https://www.un-ilibrary.org/content/books/9789210024914.

